# Oligomer-Targeting Prevention of Neurodegenerative Dementia by Intranasal Rifampicin and Resveratrol Combination – A Preclinical Study in Model Mice

**DOI:** 10.3389/fnins.2021.763476

**Published:** 2021-12-13

**Authors:** Tomohiro Umeda, Ayumi Sakai, Keiko Shigemori, Ayumi Yokota, Toru Kumagai, Takami Tomiyama

**Affiliations:** ^1^Department of Translational Neuroscience, Osaka City University Graduate School of Medicine, Osaka, Japan; ^2^Medilabo RFP, Inc., Kyoto, Japan

**Keywords:** rifampicin, resveratrol, intranasal administration, Aβ, tau, α-synuclein, oligomer, prevention

## Abstract

Amyloidogenic protein oligomers are thought to play an important role in the pathogenesis of neurodegenerative dementia, including Alzheimer’s disease, frontotemporal dementia, and dementia with Lewy bodies. Previously we demonstrated that oral or intranasal rifampicin improved the cognition of APP-, tau-, and α-synuclein-transgenic mice by reducing the amount of Aβ, tau, and α-synuclein oligomers in the brain. In the present study, to explore more effective and safer medications for dementia, we tested the drug combination of rifampicin and resveratrol, which is a multifunctional natural polyphenol with the potential to antagonize the adverse effects of rifampicin. The mixture was intranasally administered to APP-, tau-, and α-synuclein-transgenic mice, and their memory and oligomer-related pathologies were evaluated. Compared with rifampicin and resveratrol alone, the combinatorial medicine significantly improved mouse cognition, reduced amyloid oligomer accumulation, and recovered synaptophysin levels in the hippocampus. The plasma levels of liver enzymes, which reflect hepatic injury and normally increase by rifampicin treatment, remained normal by the combination treatment. Notably, resveratrol alone and the combinatorial medicine, but not rifampicin alone, enhanced the levels of brain-derived neurotrophic factor (BDNF) and its precursor, pro-BDNF, in the hippocampus. Furthermore, the combination showed a synergistic effect in ameliorating mouse cognition. These results show the advantages of this combinatorial medicine with regards to safety and effectiveness over single-drug rifampicin. Our findings may provide a feasible means for the prevention of neurodegenerative dementia that targets toxic oligomers.

## Introduction

Neurodegenerative dementia is defined as neurodegenerative diseases with a main clinical symptom of dementia, which includes Alzheimer’s disease (AD), frontotemporal dementia (FTD), and dementia with Lewy bodies (DLB). Parkinson’s disease (PD) is also known to pose dementia when its pathologies spread into the cerebral cortex. These disorders are characterized by the cerebral accumulation of disease-specific amyloidogenic proteins: Aβ and tau in AD, tau or TDP-43 in FTD, and α-synuclein in DLB and PD. These proteins tend to self-aggregate into insoluble fibrils with the β-sheet structure leading to the formation of characteristic pathological inclusions in the brain, such as senile plaques composed of Aβ, neurofibrillary tangles of hyperphosphorylated tau, and Lewy bodies of phosphorylated α-synuclein. While these inclusions are helpful for the differential diagnosis of neurodegenerative diseases, accumulating evidence indicates that the real causal culprit of the disease is smaller, soluble oligomers of the proteins. For example, synaptic dysfunction, which is assumed an immediate early symptom in AD, is caused by soluble Aβ oligomers (Cline et al., 2018; Li and Selkoe, 2020), and Aβ oligomers trigger the pathological cascade of AD including tau hyperphosphorylation, glial activation, and neuronal loss (Tomiyama et al., 2010; Cline et al., 2018). Tau oligomers (Maeda and Takashima, 2019; Hill et al., 2020) and α-synuclein oligomers (Bengoa-Vergniory et al., 2017; Kayed et al., 2020) are also suggested to play a crucial role in the pathogenesis of tauopathy and α-synucleinopathy, respectively.

Despite the vigorous efforts of researchers and pharmaceutical companies, there is no effective cure for neurodegenerative dementia. Many drug candidates have been developed, but most have failed to show beneficial effects on patients’ cognition in clinical trials. These failures are primarily attributed to two main reasons: the late timing of medication and the wrong drug target. The treatment should be started early, before the neurodegeneration proceeds, and the drug target should be set to the toxic oligomers. We previously demonstrated that a well-known antibiotic, rifampicin, inhibited the oligomerization of Aβ, tau, and α-synuclein *in vitro* and that the activity was specific to pathological amyloidogenic proteins but not to physiologically assembling proteins (Umeda et al., 2016). Furthermore, when orally administered to APP- and tau-transgenic (Tg) mice, rifampicin reduced Aβ and tau oligomers in the brain and improved the cognition of the mice (Umeda et al., 2016). These results suggest that rifampicin is a promising oligomer-targeting medicine and can prevent neurodegenerative dementia when administered early before the neurodegeneration.

However, oral rifampicin occasionally induces adverse effects such as liver dysfunction and drug-drug interaction (Drugs.com website^1^). Such adverse events are considered to occur during the first pass of rifampicin from the intestine to the liver. To avoid this pathway, we sought a different administration route. We noted that the drug injected into the nasal cavity is efficiently delivered to the brain by passing through the nasal mucosa epithelium and transported less to the liver (Erdõ et al., 2018). Thus, we compared three administration routes: oral, intranasal, and subcutaneous, on the safety, brain delivery, and therapeutic efficacy in APP-Tg mice (Umeda et al., 2018). Intranasal administration showed the highest brain delivery and therapeutic efficacy and improved safety. Taking the treatment invasiveness into consideration, we concluded that intranasal administration was the best way for long-term rifampicin treatment. Thereafter, we expanded our study to α-synuclein-Tg mice to show that nasal rifampicin reduced brain α-synuclein oligomers and improved mouse cognition (Umeda et al., 2021).

In the prevention of neurodegenerative dementia, drug treatment is continued for a long period, and therefore, preventive medicines must be extremely safe. To further secure the safety of nasal rifampicin, we hypothesized that rifampicin’s undesired actions could be antagonized by other compounds. Thus, we explored the literature for a compound that possesses hepatoprotective actions opposite to rifampicin and, if possible, additional clinical effects that rifampicin does not show. We consequently selected *trans*-resveratrol as a candidate. This compound is a stilbene-type natural polyphenol and a phytochemical highly contained in grapes and berries. Resveratrol is a multifunctional agent that shows antioxidant, anti-inflammatory, anti-diabetes, anti-cancer, and neuroprotective effects (Meng et al., 2020; Rahman et al., 2020). It shows high tolerability and low toxicity in humans and is widely used as a dietary supplement. Furthermore, *trans*-resveratrol inhibits the expression of cytochrome P450 3A4 (CYP3A4) and P-glycoprotein (Deng et al., 2014; El-Readi et al., 2019), both of which are enhanced by rifampicin (Kim et al., 2008; Tan et al., 2016), implying that resveratrol can neutralize the hepatotoxicity and drug-drug interaction of rifampicin. In addition, resveratrol increases the expression of brain-derived neurotrophic factor (BDNF) in animals (Shen et al., 2018; Chen et al., 2021), an effect not seen with rifampicin. BDNF plays an important role in synaptic plasticity and memory, and its levels are reduced in the brains of patients with neurodegenerative and psychiatric disorders (Miranda et al., 2019). However, resveratrol has a pharmacokinetic problem: due to its extensive glucuronidation and sulfation in the intestine and liver, the bioavailability of oral resveratrol is very low (Cottart et al., 2014). Thus, in the present study, we tested the intranasal combination of rifampicin and resveratrol in multiple mouse models of neurodegenerative dementia. The results show several advantages of this combinatorial medicine in terms of safety and effectiveness over single-drug rifampicin.

## Materials and Methods

### Mice

Four kinds of Tg mice were used. APP_OSK_ and tau784 mice were generated in our laboratory, APP23 mice were kindly provided by Novartis Pharma, Inc., and Huα-Syn (A53T) line G2-3 mice, simply referred to as αSyn-Tg mice here, were purchased from the Jackson Laboratory (Bar Harbor, ME, United States). APP_OSK_ mice express human APP695 with the Osaka (E693Δ) mutation under the mouse prion protein promoter and show an intraneuronal accumulation of Aβ oligomers and memory impairment at 8 months without forming amyloid plaques (Tomiyama et al., 2010). APP23 mice express human APP751 with the Swedish (KM670/671NL) mutation under the mouse Thy-1 promoter (Sturchler-Pierrat et al., 1997) and exhibit cognitive decline at 3 months and amyloid plaques at 6 months (Van Dam et al., 2003). Tau784 mice express both wild-type 3-repeat and 4-repeat human tau (tau410 and tau441) under the mouse CaMKIIa promoter with a dominant expression of 4-repeat human tau at adult ages by the presence of tau intron 10 + 16C-T mutation in the transgene (Umeda et al., 2013). They develop tau hyperphosphorylation, tau oligomer accumulation, and cognitive dysfunction at 6 months and neurofibrillary tangles at 15 months (Umeda et al., 2015). αSyn-Tg mice express A53T-mutant human α-synuclein under the mouse prion protein promoter (Lee et al., 2002) and display α-synuclein oligomer accumulation and cognitive impairment at 6 months and motor dysfunction at 9 months (Umeda et al., 2021). All Tg mice were maintained and used as heterozygotes.

### Rifampicin and Resveratrol Treatment

Administration of rifampicin and resveratrol to mice was started at an age at which oligomer pathology and cognitive dysfunction progressed enough. We assume that the pathological stage in model mice at the age we chose corresponds to the preclinical to prodromal stage of neurodegenerative dementia in humans. APP_OSK_ mice 13-months old were divided into 5 groups containing 5–6 male and 4–6 female each based on the drug treatment: rifampicin alone (0.02 mg/day), low-dose resveratrol alone (0.02 mg/day), high-dose resveratrol alone (0.1 mg/day), combinatorial medicine (0.02 mg rifampicin + 0.02 mg resveratrol/day), and carboxymethylcellulose (CMC) as a control. APP23 mice at 16–17-months old were divided into 2 groups containing 5–6 male and 3–4 female each: one for combinatorial medicine (0.02 mg each drug/day) and the other for CMC. Tau784 mice 13–14-months old were also divided into 5 groups containing 5–7 male and 3–5 female each: rifampicin alone (0.02 mg/day), resveratrol alone (0.02 mg/day), low-dose combinatorial medicine (0.01 mg each drug/day), high-dose combinatorial medicine (0.02 mg each drug/day), and CMC. αSyn-Tg mice 6-months old were divided into 3 groups containing 3–4 male and 5–8 female each: low-dose combinatorial medicine (0.01 mg each drug/day), high-dose combinatorial medicine (0.02 mg each drug/day), and CMC. Rifampicin (Sigma-Aldrich, St. Luis, MO, United States) and *trans*-resveratrol (Sigma-Aldrich) were dissolved to 2 or 4 mg/mL in 0.5% low-viscosity CMC (Sigma-Aldrich). Equal volumes of the rifampicin and resveratrol solutions were mixed to obtain the combinatorial medicine containing the two drugs at 1 or 2 mg/mL each. In some experiments, resveratrol solution was made at 10 mg/mL. 10 μL of rifampicin (i.e., 0.02 mg), resveratrol (0.02 or 0.1 mg), combinatorial medicine (0.01 or 0.02 mg each drug), or CMC solution was administered into the bilateral nasal cavity using microtips 5 days a week from Monday to Friday over 1 month. Age-matched non-Tg littermates were treated with CMC alone.

### Behavioral Tests

Following 1-month drug treatment, the cognitive function of the mice was evaluated by the Morris water maze test as described previously (Umeda et al., 2015, 2021). Mice were trained to swim to a hidden platform 5 times a day at 5-min intervals over 4 consecutive days. The time when mice climbed on the platform was recorded. The mean time of the 5 trials was calculated each day. Drug treatment was continued during the behavioral test. At day 5, the retention of spatial reference memory was assessed by a probe trial consisting of a 60 s free swim in the pool without the platform.

### Hepatotoxicity Assay

Hepatotoxicity of the drugs was assessed by measuring the plasma levels of liver enzymes. After the water maze task, blood samples of APP_OSK_ mice were collected under anesthesia, and the plasma was separated by centrifugation. The levels of aspartate transaminase (AST) and alanine transaminase (ALT) were measured using a 7180 Clinical Analyzer (Hitachi High-Technologies, Tokyo, Japan) with L-type Wako AST-J2 and ALT-J2 reagents (Wako Pure Chemical Industries, Osaka, Japan).

### Immunohistochemical Analysis

After the water maze task, each group was divided into two groups: one group for immunohistochemical analysis and the other for biochemical analysis. Brain sections of Tg and non-Tg littermates were prepared as described previously (Tomiyama et al., 2010). To expose the antigens, sections were boiled in 0.01 N HCl, pH2 for 10 min (for Aβ oligomers, amyloid plaques, tau oligomers, α-synuclein oligomers, and BDNF) or 10 mM citrate buffer, pH6 for 30 min (for synaptophysin). After blocking with 10% calf serum overnight, the sections were stained with antibodies for pan-Aβ (β001) (Tomiyama et al., 2010), Aβ oligomers (11A1; IBL, Fujioka, Japan), tau oligomers (TOMA1; Merck-Millipore, Darmstadt, Germany), α-synuclein oligomers (Syn33; Merck-Millipore), synaptophysin (SVP-38; Sigma-Aldrich), and BDNF (BDNF-#9; DSHB, Iowa City, IA, United States) essentially as described previously (Tomiyama et al., 2010). The staining was followed by biotin-labeled second antibody (Vector Laboratories, Burlingame, CA, United States), horseradish peroxidase (HRP)-conjugated avidin-biotin complex (Vector Laboratories), and a HRP substrate, diaminobenzidine (DAB; Dojindo, Kumamoto, Japan). For synaptophysin, immunoreactivity was visualized with FITC-labeled second antibody (Jackson Laboratory). The stained specimens were viewed under a BZ-X800 fluorescence microscope (Keyence, Osaka, Japan), and the images were collected. Aβ, tau, α-synuclein pathologies, synapse loss, and BDNF expression were evaluated by quantifying the staining intensity or area in a constant region in each image using NIH ImageJ software.

### Western Blot Analysis of BDNF

Hippocampal tissues were dissected from the brains and homogenized by sonication in 10 volumes of Tris-buffered saline containing protease inhibitor cocktail (P8340; Sigma-Aldrich). The homogenates were centrifuged at 100,000 × *g* for 30 min to separate from insoluble materials. The supernatants were subjected to western blotting with antibodies for BDNF (H-117; Santa Cruz Biotechnology, Dallas, TX, United States) and actin (Sigma-Aldrich) followed by HRP-labeled second antibodies (Bio-Rad Laboratories, Hercules, CA, United States) and a chemiluminescent substrate for HRP (ImmunoStar LD; Fujifilm-Wako, Osaka, Japan). The stained proteins were visualized and quantified using an ImageQuant LAS 500 image analyzer (GE Healthcare, Hino, Japan).

### Statistical Analysis

All experiments and data analyses were performed under unblinded conditions. Comparisons of means among more than two groups were performed using ANOVA or two-factor repeated measures ANOVA (for the Morris water maze acquisition test), followed by Fisher’s PLSD test. In all the behavioral tests we performed the interactions between group and day were not significant, and thus only the group effects were analyzed. Differences with a *P*-value of <0.05 were considered significant.

## Results

To explore more effective and safer medicines than rifampicin alone, we tested the therapeutic effects of rifampicin and resveratrol combination using four kinds of mouse models of neurodegenerative dementia.

**FIGURE 1:**
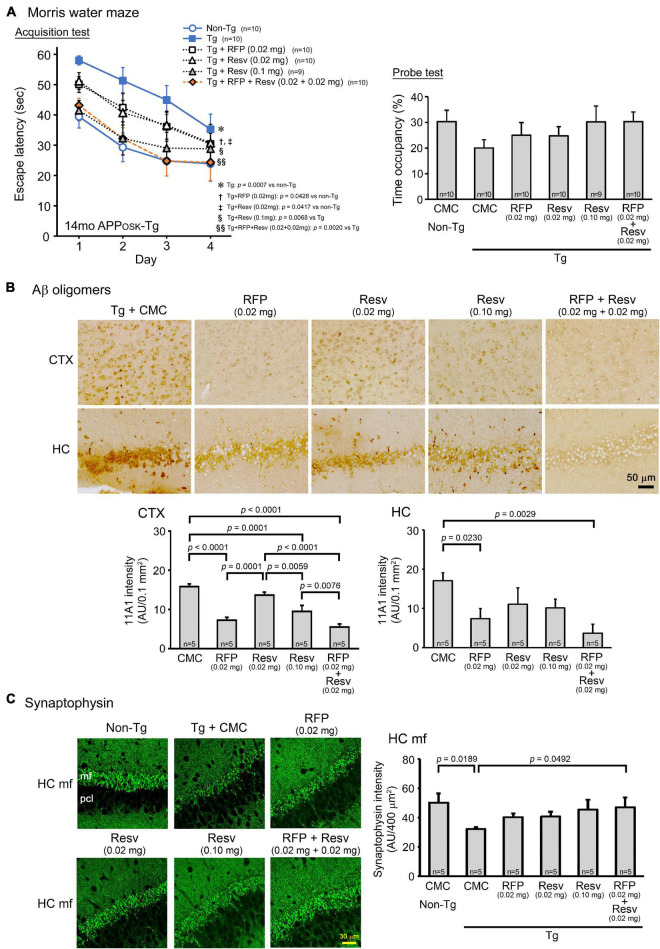
Effects of intranasal rifampicin and resveratrol combination on memory and Aβ oligomer-related pathologies in APP_OSK_ mice. Drug solution containing 0.02 mg rifampicin (RFP) alone, 0.02 or 0.1 mg resveratrol (Resv) alone, 0.04 mg combinatorial medicine (0.02 mg each drug), or carboxymethylcellulose (CMC) as a control was intranasally administered every day into 13-month-old mice for 1 month. **(A)** In the Morris water maze acquisition test, the combinatorial medicine and high-dose resveratrol showed a significant improvement of mouse memory to a level similar of non-Tg littermates. **(B,C)** After the water maze task, brain sections were stained with antibodies for Aβ oligomers (11A1) and synaptophysin. **(B)** The levels of Aβ oligomers in the cerebral cortex (CTX) and hippocampus (HC) were significantly attenuated by rifampicin alone and the combinatorial medicine. **(C)** The levels of synaptophysin in the hippocampal mossy fibers (HC mf) were significantly recovered by the combinatorial medicine to a level similar of non-Tg littermates. pcl, pyramidal cell layer. AU, arbitrary unit. Values represent the mean ± SEM.

The first model we used was APP_OSK_ mice, which show Aβ oligomer accumulation and memory impairment at 8 months but not senile plaques even at 24 months (Tomiyama et al., 2010). Drug solution containing 0.02 mg rifampicin alone, 0.02 mg resveratrol alone, or 0.04 mg combinatorial medicine (0.02 mg rifampicin + 0.02 mg resveratrol) was intranasally administered every day into 13-month-old mice for 1 month. The spatial reference memory was assessed by the Morris water maze test. Both rifampicin alone and resveratrol alone at 0.02 mg/day appeared to affect mouse memory, but their effects were not significant (Figure 1A). In contrast, the combination of the two drugs showed a significant improvement of memory, as indicated by the test performance nearing that of non-Tg littermates (Figure 1A). Probe test results appeared to support this conclusion, although no significant differences were detected between groups. We previously reported that nasal rifampicin improved the memory of 11-month-old APP_OSK_ mice almost completely at 0.05 mg/day (Umeda et al., 2018). The present results suggest that the combinatorial medicine achieved a sufficient recovery of mouse memory even at a lower dose of rifampicin than rifampicin used alone. This observation implies that by combining with resveratrol, we can lower the risk of rifampicin’s adverse effects without affecting the therapeutic efficacy. To further evaluate the therapeutic ability of resveratrol, we tested 0.1 mg resveratrol alone per day at the same time. Resveratrol at this dose showed a significant improvement of memory to a level similar of non-Tg littermates, but the effect was incomplete at days 3 and 4. Then we evaluated Aβ oligomer-related pathologies by immunohistochemistry. The levels of Aβ oligomers in the cerebral cortex and hippocampus were significantly attenuated by rifampicin alone and the combinatorial medicine (Figure 1B). Resveratrol alone also diminished the levels, but the effect was weaker even at high dose than that of rifampicin. Rifampicin alone and resveratrol alone at 0.02 mg/day recovered synaptophysin levels in the hippocampal mossy fibers, but again the effects were incomplete (Figure 1C). In contrast, the combination and high-dose resveratrol restored synaptophysin levels to a level similar of non-Tg littermates. These results imply that mouse memory reflects synaptophysin levels rather than the Aβ pathology itself.

**FIGURE 2:**
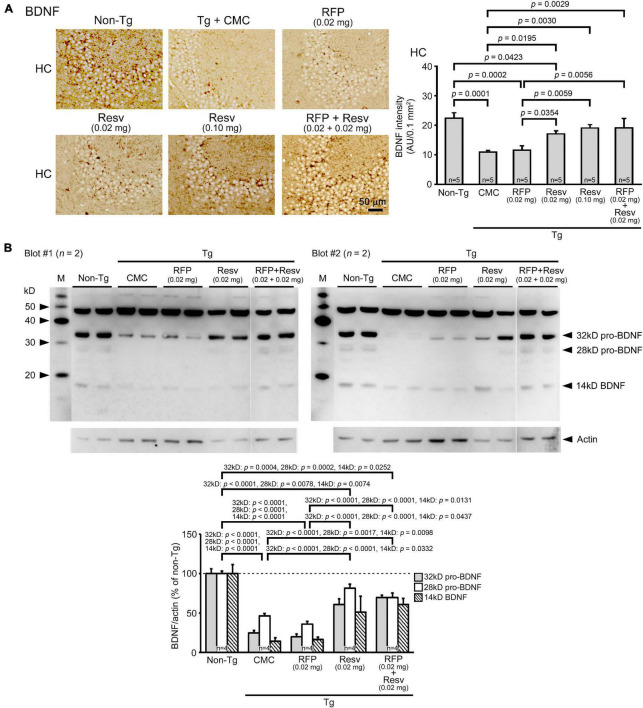
Effects of intranasal rifampicin and resveratrol combination on BDNF levels in APP_OSK_ mice. **(A)** Brain sections were stained with an anti-BDNF antibody. The level of BDNF in the hippocampal CA2/3 region was decreased in Tg mice. Resveratrol (Resv) alone and the combinatorial medicine, but not rifampicin (RFP) alone, recovered the BDNF level. **(B)** Hippocampal soluble fractions were subjected to western blotting with an antibody for BDNF. The levels of BDNF, pro-BDNF, and truncated pro-BDNF were significantly restored by Resv alone and the combinatorial medicine, but not by RFP alone. CMC, carboxymethylcellulose. M, MagicMark XP Western Protein Standard (Invitrogen, Carlsbad, CA, United States). AU, arbitrary unit. Values represent the mean ± SEM.

It has been shown that the levels of BDNF are reduced in the brain of patients with neurodegenerative and psychiatric disorders (Miranda et al., 2019). Resveratrol can induce the expression of BDNF (Shen et al., 2018; Chen et al., 2021). Thus, we examined BDNF levels in brain sections by immunohistochemical staining and in hippocampal tissues by western blot. Immunohistochemistry analysis found that APP_OSK_ mice exhibit reduced levels of BDNF in the hippocampal CA2/3 region (Figure 2A). Resveratrol alone restored the levels dose-dependently and to a level similar of non-Tg littermates at 0.1 mg/day. In contrast, rifampicin alone did not significantly affect BDNF levels. The combinatorial medicine recovered BDNF levels similarly to high-dose resveratrol. These results were confirmed by western blots. Hippocampal whole tissues were collected and their soluble fractions were analyzed. The levels of BDNF (14 kD), its precursor pro-BDNF (32 kD), and a truncated form of pro-BDNF (28 kD) (Mowla et al., 2001) were significantly decreased in APP_OSK_ mice (Figure 2B). Resveratrol alone (0.02 mg/day) and the combinatorial medicine, but not rifampicin alone, recovered the levels significantly.

To confirm the safety of the combinatorial medicine, we measured the plasma levels of AST and ALT after the behavioral test. Rifampicin-treated mice showed a slight increase in AST levels but almost no change in ALT levels (Table 1). This toxicity by rifampicin was weaker than that reported in our previous study (Umeda et al., 2018), probably because the rifampicin dose in the present study was lower (0.02 mg/day vs. 0.05 mg/day). In contrast to rifampicin, resveratrol alone did not affect AST levels at 0.02 mg/day but reduced them at 0.1 mg/day. When these two drugs were combined, the rifampicin-induced AST increase vanished, indicating that resveratrol acts on hepatocytes protectively and neutralizes rifampicin’s hepatotoxicity, as we expected.

**TABLE 1:** Plasma levels of liver enzymes in APP_OSK_ mice after 1-month intranasal administration of rifampicin, resveratrol, and their combination.

	Non-Tg mice	Tg mice				
	
	CMC	CMC	Rifampicin	Resveratrol	Resveratrol	Combination
			0.02 mg/day	0.02 mg/day	0.1 mg/day	0.02 mg each/day
	*n* = 10	*n* = 10	*n* = 10	*n* = 10	*n* = 9	*n* = 10
AST (IU/L)	99.5 ± 12.2	104.4 ± 8.3	117.6 ± 15.4	103.7 ± 9.8	86.0 ± 12.2	107.7 ± 18.2
ALT (IU/L)	30.9 ± 3.7	34.1 ± 2.9	34.4 ± 3.5	36.9 ± 4.6	29.1 ± 12.2	37.2 ± 4.2

*AST, aspartate transaminase; ALT, alanine transaminase; CMC, carboxymethylcellulose. Values represent the mean ± SEM.*

**FIGURE 3:**
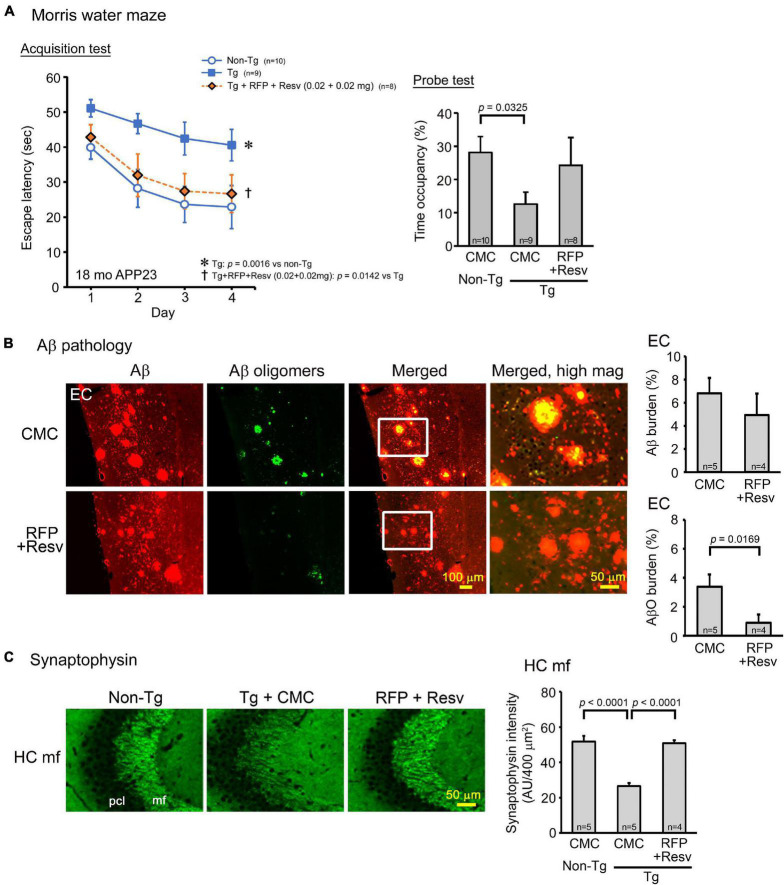
Effects of intranasal rifampicin and resveratrol combination on memory and Aβ pathologies in APP23 mice. Drug solution containing 0.04 mg combinatorial medicine (0.02 mg each drug) or carboxymethylcellulose (CMC) was intranasally administered every day into 16–17-month-old mice for 1 month. **(A)** In the Morris water maze acquisition test, the combinatorial medicine showed a significant improvement of mouse memory to a level similar of non-Tg littermates. **(B,C)** After the water maze task, brain sections were stained with antibodies for Aβ oligomers (11A1), pan-Aβ, and synaptophysin. **(B)** The level of amyloid plaques in the entorhinal cortex (EC) did not change by the treatment, whereas the level of Aβ oligomers (AβO) was significantly reduced. **(C)** The level of synaptophysin in the hippocampal mossy fibers (HC mf) was significantly recovered by the treatment to almost the same level as non-Tg littermates. pcl, pyramidal cell layer. AU, arbitrary unit. Values represent the mean ± SEM.

The second model, APP23 mice, which are a typical model of AD, show memory impairment at 3 months and amyloid plaque formation at 6 months (Van Dam et al., 2003). Drug solution containing 0.04 mg combinatorial medicine (0.02 mg each drug) was intranasally administered every day into 15-month-old mice for 1 month. In the Morris water maze, the combination showed a significant improvement of mouse memory to a level similar of non-Tg littermates (Figure 3A). Again, probe test results appeared to support this observation, although the treatment effect was not statistically significant. Immunohistochemical examination revealed that the level of amyloid plaques in the entorhinal cortex was not significantly changed by the treatment, whereas the level of Aβ oligomers was markedly reduced (Figure 3B). These results are consistent with our previous finding that rifampicin reduces Aβ oligomers but not amyloid plaques in Tg2576 mice (Umeda et al., 2016) and imply that not amyloid plaques but Aβ oligomers are closely associated with cognitive dysfunction in AD. In addition, the combinatorial medicine significantly recovered synaptophysin levels in the hippocampal mossy fibers to almost the same level as in non-Tg littermates (Figure 3C).

**FIGURE 4:**
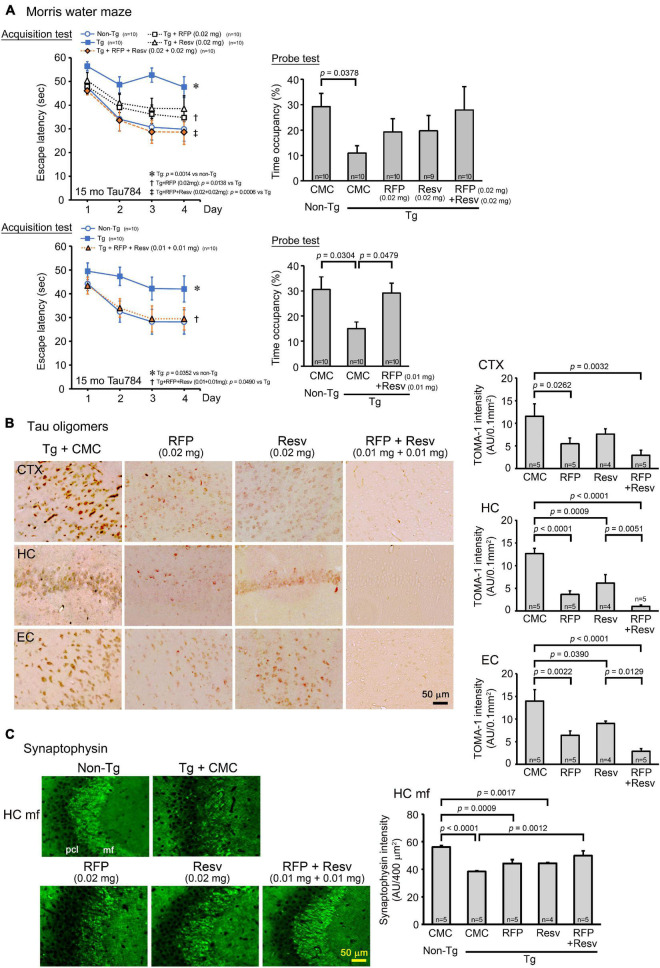
Effects of intranasal rifampicin and resveratrol combination on memory and tau oligomer-related pathologies in tau784 mice. Drug solution containing 0.02 mg rifampicin (RFP) alone, 0.02 mg resveratrol (Resv) alone, 0.02 or 0.04 mg combinatorial medicine (0.01 or 0.02 mg each drug), or carboxymethylcellulose (CMC) was intranasally administered every day into 13–14-month-old mice for 1 month. **(A)** In the Morris water maze, the combinatorial medicine showed a significant improvement of mouse memory to almost the same level as non-Tg littermates even at the lower dose. This suggests a synergistic effect of rifampicin and resveratrol in ameliorating mouse cognition. **(B,C)** After the water maze task, brain sections were stained with antibodies for tau oligomers (TOMA-1) and synaptophysin. **(B)** The levels of tau oligomers in the cerebral cortex (CTX), hippocampus (HC), and entorhinal cortex (EC) were significantly attenuated by rifampicin alone and low-dose (0.02 mg/day) combinatorial medicine. **(C)** The level of synaptophysin in the hippocampal mossy fibers (HC mf) was significantly recovered by the combinatorial medicine (0.02 mg/day) to a level similar of non-Tg littermates. pcl, pyramidal cell layer. AU, arbitrary unit. Values in each figure represent the mean ± SEM.

The third model we used was a model of FTD, tau784 mice, which show tau oligomer accumulation and memory impairment at 6 months (Umeda et al., 2015). Drug solution containing either 0.02 mg rifampicin alone, 0.02 mg resveratrol alone, or 0.04 mg combinatorial medicine (0.02 mg each drug) was intranasally administered every day into 14–15-month-old mice for 1 month. In the Morris water maze, both rifampicin alone and resveratrol alone at 0.02 mg/day appeared to improve mouse memory, but the effect was significant only in rifampicin (Figure 4A). In contrast, the combination of the two drugs (0.02 mg each drug) showed a significant improvement of memory to the same level as non-Tg littermates. We repeated this experiment with a low-dose combinatorial medicine (0.01 mg each drug). Even at the lower dose, the combination showed a complete recovery of mouse memory. This finding suggests a synergistic effect of the two drugs, although the differences between rifampicin alone (0.02 mg) and the higher dose of the combination (0.02 mg each drug) were not significant in both the acquisition and probe tests. Immunohistochemical examination revealed that the levels of tau oligomers in the cerebral cortex, hippocampus, and entorhinal cortex were significantly reduced by rifampicin alone and the low-dose combinatorial medicine (Figure 4B). Resveratrol alone also attenuated the levels, but the effects were significant only in the hippocampus and entorhinal cortex. Images revealed that the effects of rifampicin alone and resveratrol alone on tau oligomers appeared incomplete, whereas that of the combination was almost complete. This observation supports the synergistic effect in the behavioral test. Our results in Figures 1B, 4B collectively indicate that the anti-oligomer activity of rifampicin is stronger than that of resveratrol. Finally, the level of synaptophysin in the hippocampal mossy fibers was recovered by these treatments (Figure 4C). Again, the effects of rifampicin alone and resveratrol alone were not significant, but the level with the combinatorial medicine (0.02 mg/day) was similar to that of non-Tg littermates.

**FIGURE 5:**
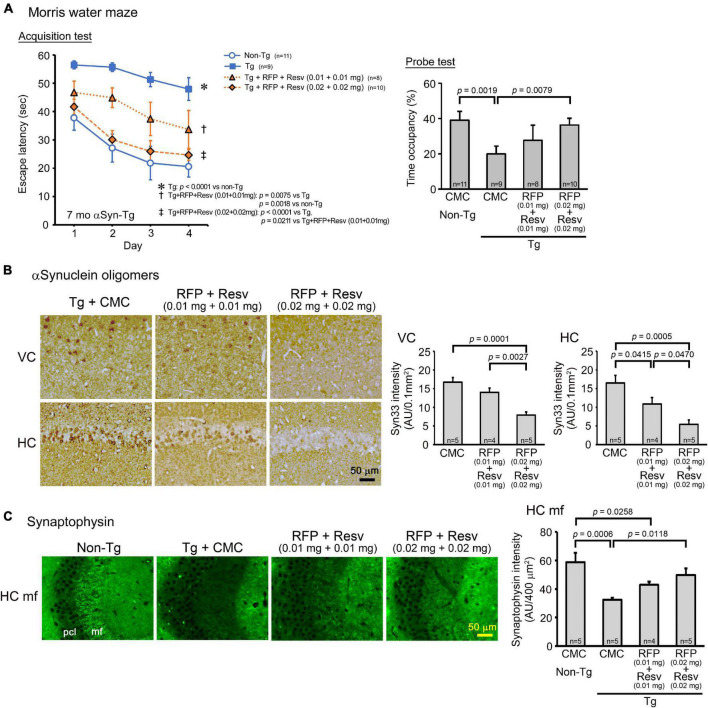
Effects of intranasal rifampicin and resveratrol combination on memory and α-synuclein oligomer-related pathologies in αSyn-Tg mice. Drug solution containing 0.02 or 0.04 mg combinatorial medicine (0.01 or 0.02 mg each drug) or carboxymethylcellulose (CMC) was intranasally administered every day into 6-month-old mice for 1 month. **(A)** In the Morris water maze, the combinatorial medicine showed a significant improvement of mouse memory, but to reach a level similar level of non-Tg littermates required the higher dose. **(B,C)** After the water maze task, brain sections were stained with antibodies for α-synuclein oligomers (Syn33) and synaptophysin. **(B)** The levels of α-synuclein oligomers in the visual cortex (VC) and hippocampus (HC) were significantly attenuated by the combinatorial medicine at the higher dose (0.04 mg/day). **(C)** The level of synaptophysin in the hippocampal mossy fibers (HC mf) was significantly recovered by the combinatorial medicine (0.04 mg/day) to a level similar of non-Tg littermates. pcl, pyramidal cell layer. AU, arbitrary unit. Values in each figure represent the mean ± SEM.

The fourth and last model was αSyn-Tg mice, which show α-synuclein oligomer accumulation in the hippocampus and memory impairment at 6 months, but not motor dysfunction until 9 months (Umeda et al., 2021). We considered this mouse line at 6–8 months a model of DLB. Drug solutions containing 0.02 or 0.04 mg combinatorial medicine (0.01 or 0.02 mg each drug) were intranasally administered every day into 7-month-old mice for 1 month. In the Morris water maze, the combinatorial medicine showed a significant improvement of memory, but only the higher dose caused an improvement that reached a level similar of non-Tg littermates (Figure 5A). In our previous report, nasal rifampicin significantly improved mouse memory when administered to 6-month-old αSyn-Tg mice at 0.1 mg/day (Umeda et al., 2021). The present results suggest that the combinatorial medicine exhibited a sufficient recovery of mouse memory at a lower dose than rifampicin alone. Immunohistochemical examination revealed that the levels of α-synuclein oligomers in the visual cortex and hippocampus were significantly reduced by the treatments (Figure 5B). Furthermore, the levels of synaptophysin in the hippocampal mossy fibers was recovered by the treatments (Figure 5C). For both α-synuclein oligomers and synaptophysin, however, sufficient amelioration required the high dose of combination.

Taken together, these results indicate that the combination of rifampicin and resveratrol has therapeutic potential in the prevention of neurodegenerative dementia with several advantages over single-drug rifampicin in terms of safety, effectiveness, and enhanced BDNF expression.

## Discussion

Based on recent findings from clinical trials, a consensus has been established that the treatment of neurodegenerative dementia should be started earlier from the asymptomatic, prodromal stages, which are before neurons die. This means that prevention is important for the treatment of these diseases. Here, we propose five requirements of preventive medicines for neurodegenerative dementia. First, preventive medicines must be extremely safe. In prevention, drug treatment is started from the middle to old ages and lasts throughout the lifetime. Notably, several anti-Aβ vaccines and antibodies have been terminated due to their adverse events despite their success at removing senile plaques (Nicoll et al., 2019; Avgerinos et al., 2021). Second, their cost should be reasonable. Particularly in emerging countries, growing medical expenses due to increasingly aging populations are a serious problem. Third, they should be easily administered so that patients can take the drug by themselves at home without the help of medical professionals. Because preventive medicines are to be used repeatedly for many years, they should be provided in a form suitable for self-medication. Fourth, they should have a broad spectrum against amyloidogenic protein oligomers, because we cannot predict which neurodegenerative diseases we will suffer from in the future. Therefore, the drugs should act on not only Aβ but also tau and α-synuclein. Fifth, they must enter the brain and cells efficiently. Tau and α-synuclein, and in some cases Aβ, accumulate within neurons and/or glial cells to form toxic oligomers. Efficient brain and cell entry will provide sufficient clinical effects at low dose, low risk, and low cost of the drug.

To fulfill these requirements, in the present study, we combined two neuroprotective compounds, rifampicin and resveratrol, and chose the nose-to-brain route for their administration. Both drugs have been shown to have antioxidant, anti-inflammatory, and anti-amyloidogenic actions (Tomiyama et al., 1996; Bi et al., 2011; Umeda et al., 2016; Ashrafizadeh et al., 2020; Meng et al., 2020; Rahman et al., 2020), but several problems have been observed when either is used alone. The intranasal combination of the two drugs can overcome these problems and even shows several advantages over single-drug treatment. First, regarding safety, rifampicin is an antibiotic whose adverse effects are well recognized. It occasionally induces hepatic injury and drug-drug interaction by inducing CYP3A4 enzyme and P-glycoprotein (Kim et al., 2008; Tan et al., 2016). Resveratrol can antagonize these rifampicin actions by inhibiting the expression of CYP3A4 and P-glycoprotein (Deng et al., 2014; El-Readi et al., 2019). Resveratrol itself is very safe and widely used as a dietary supplement. Second, regarding cost, rifampicin is now an inexpensive generic drug, and resveratrol is also cheap. Thus, the combinatorial medicine could be provided at low cost. Third, regarding the administration, oral administration is the easiest option. However, oral rifampicin can cause adverse events, and oral resveratrol is easily metabolized into inactive forms (Cottart et al., 2014). Nasal sprays also allow patients to easily and non-invasively take these drugs. Fourth, regarding the spectrum, we previously demonstrated that rifampicin shows a wide range of anti-oligomer activity and improved cognition of mouse models of AD, FTD, and DLB (Umeda et al., 2016, 2021). In the present study, the combination of rifampicin and resveratrol also rescued cognition and oligomer-related pathologies in these mouse models. Fifth and last, regarding bioavailability in the brain, we showed that the intranasal administration of rifampicin enhanced its brain delivery (Umeda et al., 2018). Furthermore, the intracellular accumulation of tau and α-synuclein oligomers, as well as Aβ oligomers in APP_OSK_ mice, were cleared by the intranasal administration of rifampicin, resveratrol, and their combination. These results suggest that intranasal rifampicin and resveratrol can penetrate the brain and cells.

It is noteworthy that by combining rifampicin and resveratrol, more than an additive effect was observed in ameliorating mouse cognition and tau pathologies, although memory retention was not significantly rescued by the treatment. This finding implies that the combination can reduce the rifampicin dose to lower the risk of adverse events while keeping the therapeutic effects. One possible explanation of the positive interaction in their effects is that resveratrol has several actions that rifampicin does not have. For example, resveratrol induces the expression of BDNF, probably through an NAD-dependent deacetylase sirtuin-1 (SIRT1) signaling pathway (Shen et al., 2018; Chen et al., 2021). BDNF plays an important role in synaptic formation and contributes to memory function (Miranda et al., 2019). We confirmed here that resveratrol alone and the combination increased BDNF and pro-BDNF levels in mouse hippocampi, but rifampicin alone did not. In addition, resveratrol increases insulin sensitivity by activating SIRT1 and AMP-activated protein kinase (AMPK) (Dludla et al., 2020; Meng et al., 2020). Insulin has been shown to function in synaptic remodeling and neurogenesis (Mainardi et al., 2015) and protects neurons from Aβ oligomers (De Felice et al., 2009). Insulin resistance and type 2 diabetes are suggested as risk factors for AD (Wei et al., 2021). Intranasal insulin is now undergoing clinical trials in AD, mild cognitive impairment, PD, and multiple system atrophy (Alzforum website^2^). Although we have not examined the effects of resveratrol on insulin sensitivity in our mice, these features of resveratrol, at least the effect on BDNF, would compensate for rifampicin’s inability to repair injured neurons. Furthermore, resveratrol can induce autophagy *via* several signaling pathways, such as the mammalian target of rapamycin (mTOR), SIRT1, the PI3K/protein kinase B pathway (Akt), and mitogen-associated protein kinase (MAPK) (Pineda-Ramírez and Aguilera, 2021). Autophagy is a cellular waste clearance system both for the generation of materials and removal of toxic components, and its dysregulation has been observed in several neurodegenerative diseases (Malik et al., 2019). We previously reported that rifampicin also restores autophagy in mice (Umeda et al., 2016), but its mechanism likely reduced the burden on autophagy and seems to be different from that of resveratrol. This may explain the much strong effect of the combinatorial medicine in attenuating tau pathologies. The anti-oligomer actions of rifampicin and neurotrophic actions of resveratrol could synergistically function to improve mouse cognition.

Following our earlier reports (Tomiyama et al., 1994, 1996, 1997), a clinical trial with oral rifampicin and doxycycline was performed for mild to moderate AD. Unfortunately, no beneficial effects on cognition or function were observed (Molloy et al., 2013). Clinical trials with oral resveratrol were also carried out for mild to moderate AD, but again no significant clinical outcomes were observed (Turner et al., 2015; Zhu et al., 2018). These results do not necessarily indicate the ineffectiveness of either drug. We hypothesize the main reason for these failures is the late timing of the medication. A recent retrospective study in non-demented patients treated with rifampicin for mycobacterium infections indicated that oral rifampicin is effective at preventing AD, but this effect needs at least 450 mg daily for 1 year (Iizuka et al., 2017). If the treatment is started before the neurodegeneration and the drug is administered intranasally, rifampicin and resveratrol might be effective at prevention AD. Regarding the daily dose, our results show that at least 0.02 mg rifampicin and 0.02 mg resveratrol are necessary for sufficient cognitive improvement in APP23 and αSyn-Tg mice. Assuming a mouse body weight of 20 g, the 0.02 mg/day dose corresponds to 1 mg/kg a day. When we calculate the human equivalent dose based on the body surface area according to Nair and Jacob (2016), a dose of 0.081 mg/kg a day is needed. This amount is far less than the oral rifampicin daily dose for tuberculosis or leprosy (10 mg/kg a day, maximum 600 mg/day) (Drugs.com website^3^). In the present study, we combined rifampicin (MW 822.94) and resveratrol (MW 228.24) at 1:1 in weight and used CMC as the solvent for the drugs. Further investigation is necessary to determine the appropriate ratio of the two drugs and the adequate solvent for intranasal administration in patients. Pharmacokinetic and general toxicity studies of the intranasally administered drugs are also required. However, since rifampicin and resveratrol are widely used and well-known pharmaceutical and dietary supplements, respectively, we expect that the development of the combinatorial medicine should not be too challenging, which merits their drug repositioning.

In conclusion, the present results indicate that the intranasal administration of rifampicin and resveratrol combination has great advantages over single-drug rifampicin in terms of safety and effectiveness. Our findings provide a feasible means for the prevention of neurodegenerative dementia by targeting toxic oligomers.

## Data Availability Statement

The data obtained in this study are available upon reasonable request to the corresponding author.

## Ethics Statement

All animal experiments were approved by the Ethics Committee of Osaka City University and performed in accordance with the Guide for Animal Experimentation, Osaka City University.

## Author Contributions

TU performed the behavioral test, immunohistochemical staining, statistical analysis, and composed the figures. AS performed the drug administration, immunohistochemical staining, and western blotting. KS contributed to the immunohistochemical staining. AY assisted with the drug administration and the western blotting. TK proposed the drug combination study and provided financial support. TT designed the study, conducted the drug administration, immunohistochemical staining, western blotting, and wrote the manuscript. All authors approved the manuscript.

## Conflict of Interest

TU, TK, and TT have applied for a patent on intranasal rifampicin and resveratrol combination for neurodegenerative dementia (PCT/JP2019/000278). TK and TT are the founders of Medilabo RFP, Inc., who is developing nasal rifampicin. This study received funding from Medilabo RFP, Inc., The funder had the following involvement with the study: TK proposed the drug combination study, and TT designed the study, conducted the drug administration, immunohistochemical staining, western blotting, and wrote the manuscript. The remaining authors declare that the research was conducted in the absence of any commercial or financial relationships that could be construed as a potential conflict of interest.

## Publisher’s Note

All claims expressed in this article are solely those of the authors and do not necessarily represent those of their affiliated organizations, or those of the publisher, the editors and the reviewers. Any product that may be evaluated in this article, or claim that may be made by its manufacturer, is not guaranteed or endorsed by the publisher.

## Footnotes

1
https://www.drugs.com/mtm/rifampin.html

2
https://www.alzforum.org/therapeutics/nasal-insulin

3
https://www.drugs.com/dosage/rifampin.html
